# Altered CD39 and CD73 Expression in Rheumatoid Arthritis: Implications for Disease Activity and Treatment Response

**DOI:** 10.3390/biom14010001

**Published:** 2023-12-19

**Authors:** María Angels Ortiz, Cesar Diaz-Torné, Juan Jose De Agustin, Paula Estrada, Delia Reina, María Victoria Hernandez, Hye Sang, Carlos Zamora, Elisabet Cantó, Hector Corominas, Silvia Vidal

**Affiliations:** 1 Inflammatory Diseases, Institut de Recerca de l’Hospital de la Santa Creu i Sant Pau, Biomedical Research Institute Sant Pau (IIB Sant Pau), 08041 Barcelona, Spain; carlosza86@gmail.com (C.Z.); ecanto@santpau.cat (E.C.); svidal@santpau.cat (S.V.); 2 Rheumatology Department, Hospital de la Santa Creu i Sant Pau, 08041 Barcelona, Spain; cesardiaztorne@gmail.com (C.D.-T.); hsang@santpau.cat (H.S.); hcorominas@santpau.cat (H.C.); 3 Rheumatology Department, Hospital Vall d’Hebrón, 08035 Barcelona, Spain; jjagor@hotmail.com; 4 Rheumatology Department, Hospital Moisès Broggi, Sant Joan Despí, 08970 Barcelona, Spain; paulavestradaa@gmail.com (P.E.); deliareinasanz@gmail.com (D.R.); 5 Rheumatology Department, Hospital Clínic de Barcelona, 08036 Barcelona, Spain; vhernandez@medicaldoctor.hospital; 6 Department of Rheumatology, Universitat Autònoma de Barcelona, Plaça Cívica, 08193 Bellaterra, Spain

**Keywords:** rheumatoid arthritis, CD39, CD73, tocilizumab, IL-6

## Abstract

In rheumatoid arthritis (RA) synovium, ATP, and ADP are released, sparking inflammation. Ectoenzymes CD39 and CD73 metabolize these purine nucleotides, generating anti-inflammatory adenosine. Therefore, dysregulated CD39 and CD73 expression may impact RA development. We assessed CD39 and CD73 expression in peripheral blood from 15 healthy controls (Cs) and 35 RA patients at baseline and after 3 and 6 months of tocilizumab treatment using flow cytometry. Additionally, ectoenzyme expression was examined on cultured T cells to understand activation and IL-6 effects. At baseline, RA patients exhibited a lower CD8^+^CD39^−^CD73^+^ cell percentage, which inversely correlated with DAS28. Additionally, they had lower percentages of Treg CD39^+^CD73^+^ and CD39^−^CD73^−^ cells. Good responders tended to have lower B CD39^+^CD73^+^ cell percentages at baseline and 3 months. Additionally, Treg, CD8^+^ T and B cells inversely correlated with DAS28. T-cell activation increased CD39 and decreased CD73 expression, regardless of IL-6. IL-6 reduced IFNγ-secreting CD4^+^ T-cell percentage in Cs, but increased the percentage of IFNγ-secreting CD4^+^ and CD8^+^ T cells in RA patients. These findings indicate differing CD39 and CD73 expression in RA and Cs, influenced by T-cell activation and IL-6. Correlations between these molecules and RA activity suggest their role in dysregulated inflammation in RA.

## 1. Introduction

Rheumatoid arthritis (RA) is a systemic inflammatory autoimmune disorder characterized by synovial proliferation, bone destruction, and degradation of articular cartilage. The infiltration of lymphocytes and other immune cells characterizes the inflammation of the synovial membrane of RA patients. IL-6 is directly implicated in RA pathogenesis [1]. IL-6 is involved in the activation of both innate and adaptive immune cells and contributes to chronic inflammatory processes in RA. In RA patients, joint damage leads to increased levels of ATP in the synovial fluid [2].

Purinergic system components play a crucial role in the pathophysiology of RA due to their proinflammatory and anti-inflammatory actions [3]. The purinergic system comprises mediators, receptors (P1 and P2 families), transporters, and enzymes that regulate nucleoside and nucleotide levels [4]. Purinergic signaling is initiated by the release of ATP and ADP into the extracellular space through channels or transporters. Elevated levels of extracellular ATP activate P2 receptors, which often result in the production of proinflammatory cytokines and recruitment of leukocytes to damaged sites. P1 and P2 are expressed in various immune and non-immune cells, and different immunomediators modulate their density and sensitivity. Most immune cells express both of them [5]. Extracellular ATP is hydrolyzed by E-NTPDase (EC 3.6.1.5; CD39), which catalyzes the breakdown of ATP and ADP, generating AMP [6]. Subsequently, E-5′-nucleotidase (EC 3.1.3.5; CD73) hydrolyzes AMP, resulting in the production of adenosine [7]. Adenosine binds to specific P1 receptors (A1, A2A, A2B, and A3 subtypes, ARs) and typically elicits anti-inflammatory responses [8]. ARs are expressed in human synoviocytes, and A3 agonists prevent cartilage damage and bone destruction [3,8]. The alterations in purinergic receptors affect the local and systemic environment. ATP and adenosine act as signal molecules in an autocrine or paracrine manner and can implicate different pathways depending on the cell type where they act.

CD39 and CD73 are ectoenzymes expressed on the surface of immune cells, including monocytes, neutrophils, dendritic cells, myeloid-derived suppressor cells, B lymphocytes, and some T-cell subsets. Most human Treg cells are reported to express CD39, with a few of them also expressing CD73 [9]. In addition to maintaining immune homeostasis and limiting the inflammatory cascade through adenosine production, CD39 and CD73 may have other roles in immune cells, such as differentiation and cell adhesion [10,11].

Dysregulation in the expression of these two ectoenzymes has been implicated in the pathology of RA and their regulation during treatment [2,11,12,13,14]. For example, it has been shown that Treg cells from RA patients who do not respond to methotrexate treatment express lower levels of CD39 compared to healthy controls (Cs) [15]. Furthermore, T and B cells from the synovial fluid of patients with oligoarticular juvenile idiopathic arthritis (JIA) exhibit decreased CD73 expression that correlates with disease severity [16]. Thiolat et al. showed that the frequency of functionally suppressive CD39^+^ Tregs is increased as a result of anti-IL-6R treatment [17]. Given the involvement of CD39 and CD73 in the pathophysiology of RA, we analyzed the expression of these molecules on different immune cells that contribute to RA development and maintenance. This study aimed to characterize the expression of CD39 and CD73 on immune cells of RA patients before and after IL-6 blockage with tocilizumab (TCZ) treatment and to compare good responders (Rs) and non-good responders (NRs). Our results revealed distinctive CD39 and CD73 expression patterns in RA patients compared to Cs, with these expressions associated with disease activity indexes. Our findings suggest the involvement of CD39 and CD73 in RA pathophysiology, emphasizing the potential impact of T-cell activation and cytokine signaling on their expression.

## 2. Materials and Methods

### 2.1. Patient Samples and Study Design

Peripheral blood samples were obtained from 35 RA patients and 15 Cs in the Barcelona area, specifically from Hospital de la Santa Creu i Sant Pau, Hospital General de l’Hospitalet, Hospital Universitari Vall d’Hebron, and Hospital Clínic de Barcelona. The patients were diagnosed with RA based on the ACR/EULAR 2010 criteria [18]. All RA patients received TCZ as part of their treatment, as they were refractory to standard treatment with disease-modifying anti-rheumatic drugs (DMARDs), including methotrexate. TCZ treatment followed European and Spanish guidelines [19,20]. The study was approved by the ethics committee at Hospital de la Santa Creu i Sant Pau and the other participating centers. Written information about the study was provided to the patients, and they provided their consent to participate in accordance with the Declaration of Helsinki. Both the RA patients and Cs were over 18 years of age with a body weight of less than 150 kg. All RA patients exhibited moderate-to-severe disease activity (DAS28 > 3.2 ≤ 5.1 moderate and >5.1 severe) with a disease duration of 12 months or longer. In our cohort, 4 patients experienced non-serious adverse effects and TCZ was ineffective in 5 patients.

Peripheral blood (PB) samples were collected using heparin vacutainer tubes (BD, Franklin Lakes, NJ, USA), and additional PB samples were collected in EDTA tubes (BD). Blood samples and clinical data were collected before infusion at baseline (t0) and at 3 months (t3) and 6 months (t6) after initiating treatment. Patients received subcutaneous treatment with 162 mg of TCZ every week. Laboratory analysis included a hemogram and measurement of erythrocyte sedimentation rate (ESR), C-reactive protein (CRP), immunoglobulins (IgG, IgA, and IgM), rheumatoid factor (RF) and anti-cyclic citrullinated peptide (ACPA) antibodies, and was performed at each visit in all participating hospitals. Clinical data collected included DAS28, SDAI, CDAI [21] (Table 1), and treatment response according to EULAR criteria [22]. Patients who showed a good response after 6 months of treatment (EULAR = 2) were classified as good responders (Rs), while patients with no or moderate response (EULAR = 0 or 1 respectively) were classified as non-good responders (NRs). Both groups exhibited similar clinical parameters prior to treatment (Table S1).

### 2.2. Serum Analysis of RF and ACPAs

Rheumatoid factor was determined using the rate nephelometric test as per the manufacturer’s instructions (Beckman ICS II, Beckman Coulter). ACPA titers were determined by the EliA test with UniCAP (Phadia Laboratory Systems, Uppsala, Sweden).

### 2.3. Flow Cytometry

For cell phenotype analysis, monoclonal antibodies were directly added to 100 μL aliquots of heparinized whole blood and incubated at room temperature for 15 min. The antibodies used were: CD3 PECy7, CD14 APC, CD56 PE-Cy5 and CD19 PerCp (Biolegend, San Diego, CA, USA), CD4 Viogreen (Miltenyi Biotec GmBh, Bergisch Gladbach, Germany), CD39 Fitc, CD73 PE, CD25 BV421 and CD127 AlexaFluor 647 (BD Biosciences, San Jose, CA, USA). Red blood cells were lysed and white cells were fixed using BD FACS lysing solution (BD Bioscience). Negative populations were determined using respective anti-human isotype controls. Samples were acquired using a MACSQuant Analyzer 10 flow cytometer (Miltenyi Biotec GmBh) and the data were analyzed using FlowJo software v. 10.0 (TreeStar Inc., Ashland, OR, USA).

We compared the frequencies of four subsets based on CD39 and CD73 expression (CD39^−^CD73^+^, CD39^+^CD73^+^, CD39^+^CD73^−^, and CD39^−^CD73^−^) within each studied population (B cells as CD3^−^CD19^+^, CD4^+^ T cells as CD3^+^CD4^+^, CD8^+^ T cells as CD3^+^CD4^−^, NK cells as CD3^−^CD4^−^CD19^−^CD56^+^, Tregs as CD3^+^CD4^+^CD25^++^CD127^−^, and monocytes as CD14^+^). The percentage of these four subsets was related to their corresponding precursor populations (CD4^+^ T, CD8^+^ T, B, NK, and monocytes). In the case of Treg, the percentage was related to CD4^+^ T cells.

### 2.4. Luminescence Assays for Quantification of Plasmatic Levels of ATP and ADP

ADP was indirectly measured using an enzyme-coupled assay based on rapid ADP transphosphorylation with an excessive amount of exogenous UTP in the presence of NDPK. The ATP generated was quantified using an ATPLite assay kit (PerkinElmer Inc., Waltham, MA, USA) following the manufacturer’s instructions. For each sample, two wells with 20 μL of plasma from peripheral blood collected in an EDTA tube were used. One well contained 200 μM UTP and 5 U/mL NDPK (Sigma Aldrich, Burlington, MO, USA) and was labeled “B”, while the other well did not contain UTP or NDPK and was labeled “A”. The luminescence of the samples was measured using a microplate reader (Infinite M200 Pro, Tecan, Hombrechtikon, Germany). In well B, the concentration represented ATP + ADP, while in well A, it represented ATP concentration. ADP concentration was calculated by subtraction [23].

### 2.5. Cellular ATP Consumption Assay

A total of 2.5 × 10^4^ peripheral blood mononuclear cells (PBMCs)/well from C and RA patients at baseline and after treatment were incubated at room temperature for 10 min in the presence of 25 μM ATP and the ATPase inhibitor ARL67156 (Sigma-Aldrich). The ATP was quantified using the ATPLite assay kit following the manufacturer’s instructions. ATPase activity was measured by a reduction in ATP levels at the end of the assay.

### 2.6. Cytokine Levels in Serum and Supernatants

For quantification of IL-35 (Elabscience, Houston, TX, USA), IL-10 (ImmunoTools), and IL-17 (Peprotech, Cranbury, NJ, USA) concentrations, we used specific ELISAs according to the manufacturer’s instructions.

### 2.7. Cell Culture Preparation

PBMCs were prepared from RA patients before treatment and Cs using Ficoll hypaque (Lymphoprep, Axis-Shield, Oslo, Norway) density gradient centrifugation. In RA patients, the peripheral blood was collected at baseline and was from 4 R-RA and 3 NR-RA patients. PBMCs were stimulated or not with anti-CD3/CD28 beads (Gibco, Waltham, MA, USA) at a bead:PBMC ratio of 1:1. PBMCs were incubated for 72 h with or without 10 ng/mL of recombinant human IL-6 (IL-6) (ImmunoTools, Friesoythe, Germany). The PBMCs were stained with the following monoclonal antibodies: anti-human CD3-Viogreen (Miltenyi Biotec GmBh), CD39-Fitc and CD73-PE (BD Biosciences), and CD8-PECy7 (Biolegend). Samples were acquired using the MACSQuant Analyzer 10 flow cytometer, and the data were analyzed using FlowJo software.

### 2.8. Determination of IFNγ Secretion

Four hours before stopping the culture, PMA (50 ng/mL) and ionomycin (1 μg/mL) (Sigma-Aldrich) were added. After incubation, the stimulated PBMCs were collected and IFNγ secretion was determined using an IFNγ secretion assay detection kit APC (Miltenyi Biotec GmBh). Additionally, the cells were stained with the following monoclonal antibodies: anti-human CD3-Viogreen (Miltenyi Biotec GmBh), CD8-PECy7, and CD25-APCy7 (Biolegend), CD39-Fitc and CD73-PE (BD Biosciences) as well as violet-fluorescent reactive dye (Thermofisher, Waltham, MA, USA). Samples were acquired with the MACSQuant Analyzer 10 flow cytometer, and the data were analyzed using FlowJo software.

### 2.9. Statistical Analysis

Statistical analyses were performed using GraphPad Prism 8 software (v.8.0.02) and SPSS version 18.0 for Windows. The Kolmogorov–Smirnov test was used to assess the normal distribution of the data. Variables with a normal distribution are expressed as means ± SD, while variables that did not pass the Kolmogorov–Smirnov test are expressed as medians (IQR). A *t*-test (or ANOVA for more than 2 variables) and paired *t*-test (or repeated-measures ANOVA for more than 2 variables) were used for the comparison of independent and related variables with normal distribution, respectively. The Mann–Whitney test (or Kruskal–Wallis test for more than 2 variables) and the Wilcoxon signed-rank test were used for the comparison of independent and related variables with a non-normal distribution, respectively. Pearson’s coefficient was used to measure the relationship between two variables and their association. For all analyses, *p* values less than or equal to 0.05 were considered significant.

R-studio was used to plot the correlograms. The correlation coefficients (r) were denoted by the different shapes and colors of ellipses as per [24]. Thin and deeply colored ellipses indicate the strongest correlations and the inclination of an ellipse indicates the sign of the correlation (thin blue ellipses for positive correlations, thin red for negative). The correlograms were compared using the Mantel correlation test. Additionally, linear regression r-values from RA and C correlation matrices were represented by the values from correlation coefficients in Cs, considered the independent variable (x), and the correlation coefficients observed in RA patients were considered the dependent variable (y). The 95% confidence interval (CI) and 95% prediction interval (PI) were calculated using Excel 2010 by applying Student’s t statistic and considering the predicted values for RA to be equal to the values for Cs.

## 3. Results

### 3.1. Expression of CD39 and CD73 on Peripheral Blood Lymphocytes and Monocytes

RA patients presented moderate or severe disease activity, and 80% of them were positive for ACPA or RF (Table 1).

**Figure 1:**
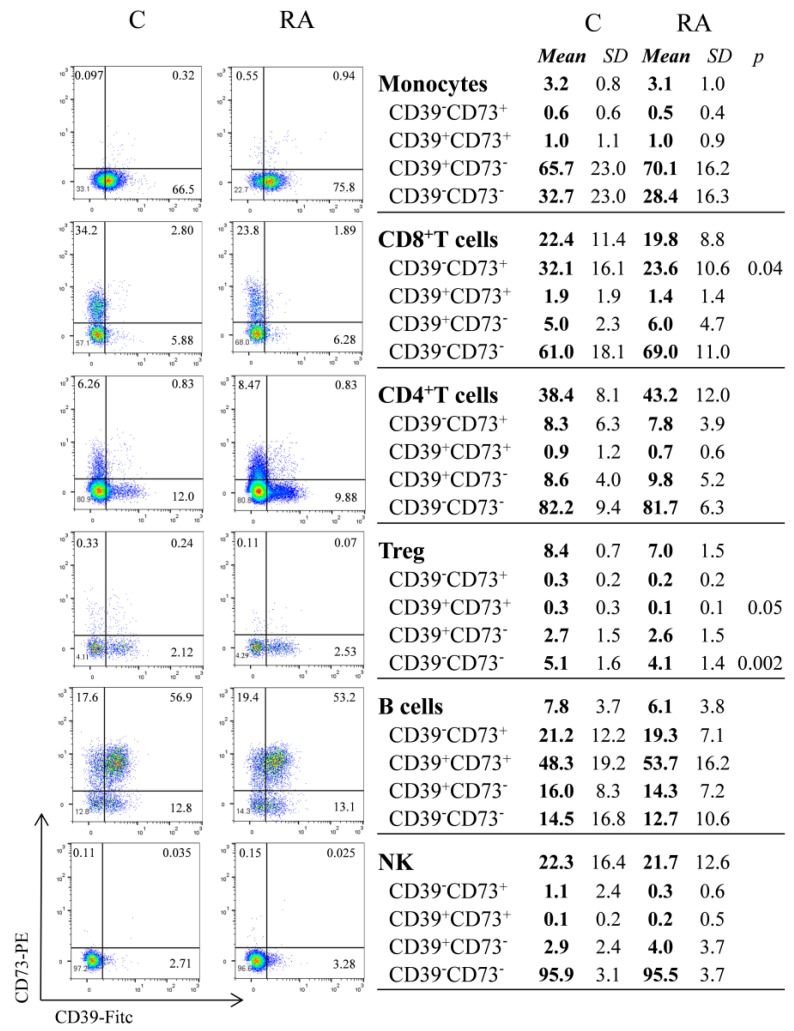
Flow cytometric analysis of CD39 and CD73 on monocytes, CD8, CD4, Treg, B, and NK cells from peripheral blood of untreated RA patients (RA) and healthy controls (C). Representative dot plots for surface expression of CD39 and CD73 and frequencies of different subpopulations (CD39^+^CD73^+^, CD39^+^CD73^−^, CD39^−^CD73^+^, and CD39^−^CD73^−^). Data are expressed as means and SD (standard deviation) of the percentage of the corresponding population (CD4^+^ T cells, CD8^+^ T cells, B cells, NK, lymphocytes, and monocytes). In the case of Treg (CD4^+^CD25^high^CD127^low^) the percentage is expressed for total CD4^+^ T cells. An unpaired *t*-test was used to compare the percentages in the RA and C groups.

We first characterized CD39 and CD73 expression on immune cells from peripheral blood samples collected from RA patients and Cs. We divided monocytes, CD8^+^ T, CD4^+^ T, Treg, B, and NK cells into four subsets based on the expression of CD39 and CD73 (Figure 1).

Approximately half the circulating B cells coexpressed CD39 and CD73, while CD8^+^ T cells predominantly expressed CD73. In contrast, monocytes, Treg cells, and NK cells predominantly expressed CD39, while CD4^+^ T cells expressed CD39 or CD73. In all cases, the frequencies were mostly comparable between RA patients and Cs. We found that the percentage of CD8^+^CD39^−^CD73^+^ cells was significantly lower in RA patients compared to Cs (*p* = 0.04). We also found that the percentage of Treg CD39^+^CD73^+^, and Treg CD39^−^CD73^−^ was lower in RA patients (*p* = 0.05 and *p* = 0.002 respectively) (Figure 1). The baseline frequency of the four different subsets was comparable among patients with or without adverse effects or ineffective treatment.

To determine the immunosuppressive potential of Tregs, we analyzed the correlation between subsets and plasmatic levels of anti-inflammatory cytokines IL-10 and IL-35. We found higher levels of IL-35 (Cs = 0.36 ± 0.4 pg/mL and RA patients = 71.23 ± 96.17 pg/mL; *p* = 0.02) and a tendency towards higher IL-10 levels in RA patients (Cs = 89.37 ± 228 pg/mL and RA = 196.7 ± 260 pg/mL). There was no correlation between cytokine concentrations and Treg subsets.

### 3.2. Relationship between CD39 and CD73 Expression with the Disease Activity Indexes

We next examined the relationships among the four subsets of CD4^+^ T cells, CD8^+^ T cells, Treg cells, B cells, and monocytes (Mo) in both RA patients at baseline and Cs using correlograms (Figure S1). We did not observe any statistically significant correlation between NK cell subsets and any of the other included subsets.

Among 43 correlation coefficient (r) values that fell outside the range defined by the upper and lower prediction intervals (PIs) (Figure S2a), we observed 30 correlations with statistical significance in at least one correlogram, which are highlighted by squares with a black border in RA patients and C correlograms in Figure S1. In these 30 correlations (Figure S2b), 3 were significant in both the C and RA patient groups, while 2 were significant exclusively in the RA patients group. Additionally, 25 correlations showed significance solely in the C group.

**Figure 2:**
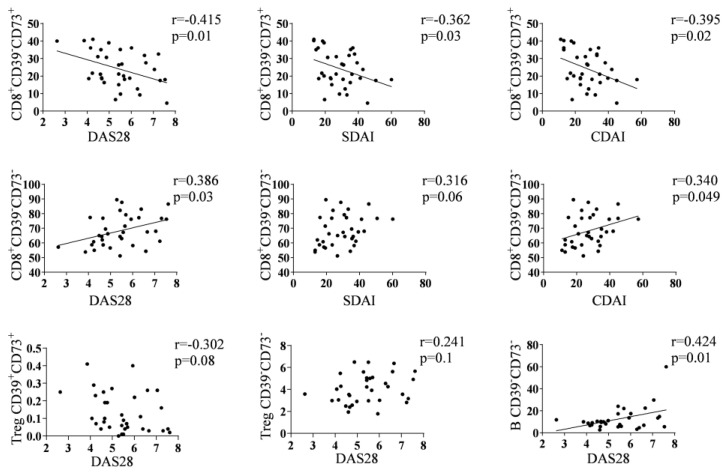
Correlations between some subsets and clinical parameters. Correlation plots corresponding to relationships between disease activity indexes and the percentages of CD8^+^CD39^−^CD73^+^, CD8^+^CD39^−^CD73^−^, Treg CD39^+^CD73^+^, Treg CD39^−^CD73^−^ or B CD39^−^CD73^−^ subsets in RA patients at baseline. Only the plots with a tendency line presented a significative correlation applying Pearson’s test.

The percentage of CD8^+^CD39^−^CD73^+^ exhibited an inverse correlation with disease activity indexes: DAS28 (r = −0.415 and *p* = 0.01), SDAI (r = −0.395 and *p* = 0.02), and CDAI (r = −0.362 and *p* = 0.03). Conversely, the percentage of CD8^+^CD39^−^CD73^−^ showed a positive correlation with DAS28 (r = 0.386 and *p* = 0.03), SDAI (r = 0.340 and *p* = 0.049) and CDAI (r = 0.316 and *p* = 0.06). Furthermore, the percentage of Treg CD39^+^CD73^+^ and Treg CD39^−^CD73^−^ cells displayed a tendency for negative (r = −0.302 and *p* = 0.08), and positive (r = 0.241 and *p* = 0.1) correlations, respectively, with DAS28. Additionally, the percentage of B CD39^−^CD73^−^ cells exhibited a positive correlation with DAS28 (r = 0.424 and *p* = 0.01) (Figure 2).

### 3.3. Expression of CD39 and CD73 in Good Responders and Non-Good Responders to TCZ Treatment

**Figure 3:**
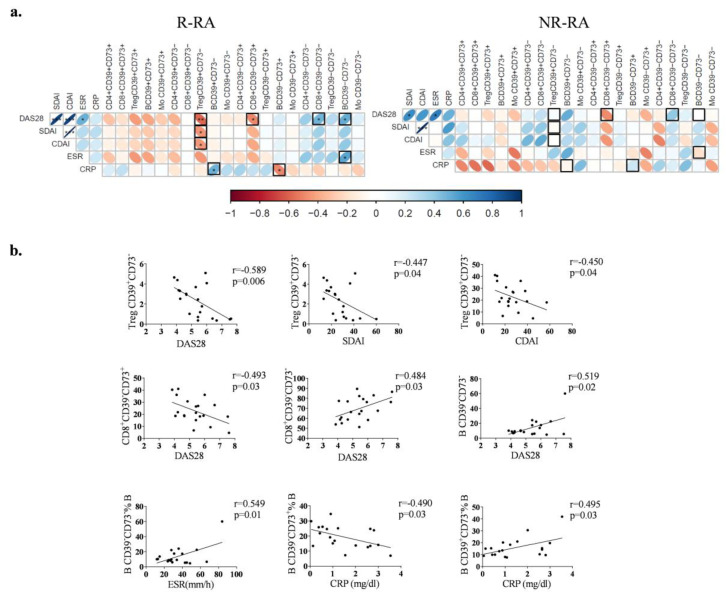
Correlations between subsets and clinical parameters (**a**) Correlograms corresponding to R-RA (n = 20) and NR-RA (n = 10) patients. Squares marked with a black border in correlogram indicate significant correlations observed in R-RA, but not in NR-RA patients. (**b**) Correlation plots illustrating the relationship between disease activity indexes and the percentage of Treg CD39^+^CD73^−^, CD8^+^CD39^−^CD73^+^, CD8^+^CD39^−^CD73^−^, B CD39^−^CD73^−^, B CD39^−^CD73^+^ or B CD39^+^CD73^−^ subsets in R-RA patients at baseline. *p* = 0.05 *, *p* = 0.01 ** and *p* = 0.001 ***.

Thereafter, the RA patients were classified into two groups: good responders (R-) and non-good responders (NR-), as outlined in Materials and Methods. Both patient groups exhibited similar clinical characteristics at baseline (Table S1). The patients who experienced non-serious adverse effects or noncomplete collection were not included in this part of the study. However, at baseline, only specific subsets within R-RA showed an association with activity indexes (Figure 3a). In R-RA patients, but not in NR-RA patients, the percentage of Treg CD39^+^CD73^−^ cells displayed a negative correlation with DAS28 (r = −0.589 and *p* = 0.006), SDAI (r = −0.447 and *p* = 0.04), and CDAI (r = −0.450 and *p* = 0.04). Likewise, the percentage of CD8^+^CD39^−^CD73^+^ cells negatively correlated with DAS28 (r = −0.493 and *p* = 0.03), while the percentage of CD8^+^CD39^−^CD73^−^ cells displayed a positive correlation with DAS28 (r = 0.484 and *p* = 0.03). Furthermore, the percentage of B CD39^−^CD73^−^ cells positively correlated with DAS28 (r = 0.519 and *p* = 0.02) as well as ESR (r = 0.549 and *p* = 0.01). Lastly, within R-RA patients, the percentage of B CD39^−^CD73^+^ cells demonstrated a negative correlation with CRP (r = −0.490 and *p* = 0.03), whereas the percentage of B CD39^+^CD73^−^ cells showed a positive correlation with CRP (r = 0.495 and *p* = 0.03) (Figure 3b).

**Figure 4:**
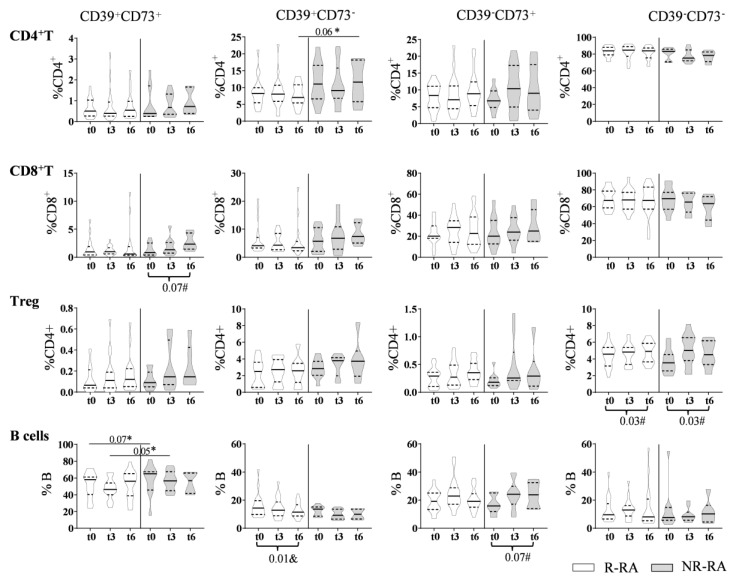
Evolution of the percentage of subsets on CD4^+^ T, CD8^+^ T, Treg and B cells in R-RA (n = 20) and NR-RA (n = 10) patients. The percentage of different subsets at baseline (t0) and after 3 or 6 months of treatment (t3 and t6, respectively) in R-RA (white violins) and NR-RA (grey violins). The *p* values in the plots correspond to comparisons between equivalent subsets in R and NR-RA applying Student’s *t*-test (*). We analyzed the changes after treatment in each subset by applying ANOVA for paired samples (#) or the Friedman test (&) (*p* values indicated at the bottom of the plots).

During the follow-up of R-RA and NR-RA patients, we found that the percentage of Treg CD39^−^CD73^−^ cells increased in both groups (*p* = 0.03), the percentage of B CD39^+^CD73^−^ cells decreased in R-RA patients only (*p* = 0.01), and the percentage of CD8^+^CD39^+^CD73^+^ cells and B CD39^−^CD73^+^ cells tended to increase in NR-RA patients (*p* = 0.07 in both). At baseline, the percentage of B CD39^+^CD73^+^ cells tended to be lower in R-RA patients compared to NR-RA patients (*p* = 0.07), and this difference became statistically significant after 3 months of treatment (*p* = 0.050). Finally, the percentage of CD4^+^CD39^+^CD73^−^ cells after 6 months of treatment tended to be lower in R-RA patients compared to NR-RA patients (*p* = 0.06) (Figure 4). We did not observe any difference in NK cell or monocyte subsets comparing R-RA and NR-RA subsets at t0, t3, or t6. Additionally, we did not observe any difference in follow-up in R-RA or NR-RA patients.

### 3.4. Plasma ATP Levels in RA Patients at Baseline and during TCZ Treatment

The differences observed at baseline in the frequencies of certain subsets based on CD39 and CD73 expression did not correspond to significant variations in the plasmatic ATP concentration between RA patients and Cs (27.51 (32.56) and 35.47 (113.73) nM, respectively). However, the ATP/ADP ratio was lower in RA patients than in Cs (1.4 (1.51) vs. 2.25 (2.08) in Cs, *p* = 0.049). Furthermore, we noted a significant decrease in ATP concentration after TCZ treatment (t0 = 27.51 (32.56), t3 = 8.69 (17.25), and t6 = 8.70 (13.05), *p* = 0.02), although the ATP/ADP ratio remained unchanged after treatment. No differences were observed between R-RA and NR-RA patients in terms of ATP concentration or ATP/ADP ratio at baseline or during the follow-up. The ATPase activity of PBMCs from RA patients, both before and after treatment, was comparable to that of the Cs. In the supernatants of PBMCs, ATP levels were similar (C = 19.86 ± 1.31 nM, RA t0 = 18.28 ± 4.97 nM, and RA t6 = 18.49 ± 3.01 nM). We did not find any correlations between ATP concentration and ATP/ADP ratios and any specific studied subset.

### 3.5. The In Vitro Effects of IL-6 on CD39 and CD73 Expression in T Cells

**Figure 5:**
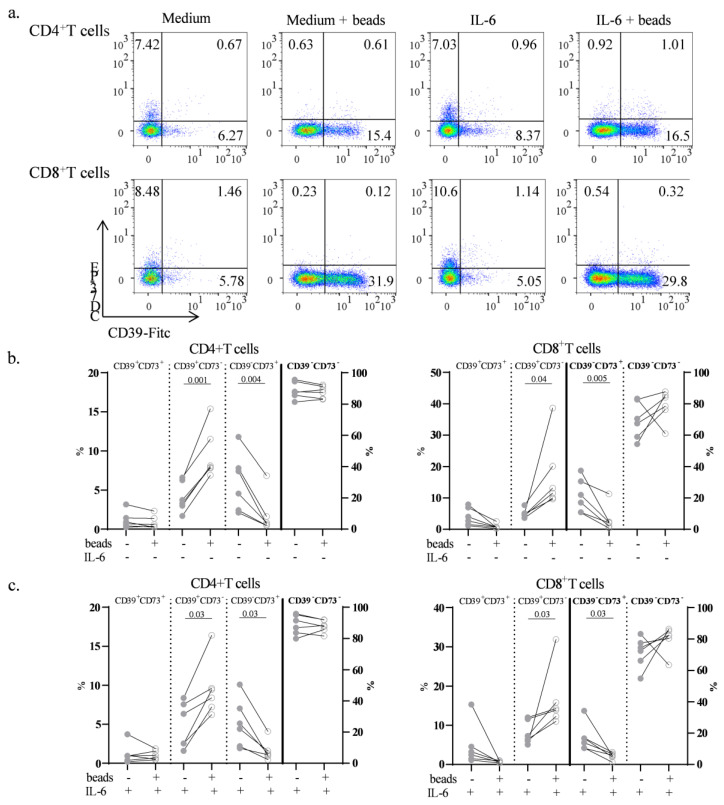
Effects of stimulation on CD39 and CD73 expression. (**a**) Representative dot plots for surface expression of CD39 and CD73 on CD4^+^ T and CD8^+^ T cells on PBMCs from Cs before culture and after 72 h cultured without beads (medium) or with beads (medium + beads). Additionally, PBMCs from Cs were cultured with IL-6, both without beads (IL-6) and with beads (IL-6 + beads). (**b**) Percentages of the four subsets of CD4^+^ T and CD8^+^ T cells from PBMCs cultured for 72 h either with anti-CD3/CD28 beads (empty dots) or without beads (full dots). (**c**) Percentages of subsets cultured under the same conditions, but with IL-6. The double-negative subset in CD4^+^ T and CD8^+^ T cells as well as the CD8^+^CD39^−^CD73^+^ subset are represented in the right axes. The Wilcoxon signed-rank test was applied in comparisons.

To mechanistically comprehend the role of IL-6 in modulating CD39 and CD73 expression, we employed an in vitro culture approach using PBMCs from Cs with TCR activation, followed by the assessment of CD39 and CD73 expression (Figure 5a). Initially, we confirmed that the expression of CD39 and CD73 T cells remained consistent both before and after culture with medium. Subsequently, we found that the activation of T cells using anti-CD3/CD28 antibodies (medium + beads) led to an increase in the CD39^+^CD73^−^ subset and a decrease in the CD39^−^CD73^+^ subset (*p* = 0.001 and *p* = 0.04, respectively) and CD8^+^ T cells (*p* = 0.04 and *p* = 0.005, respectively) (Figure 5a,b). Importantly, these changes were not influenced by IL-6, as cells cultured with IL-6 exhibited comparable outcomes to those cultured without IL-6 (Figure 5b,c). It is noteworthy that the percentage of CD8^+^CD39^−^CD73^+^ within CD8^+^ T cells cultured with IL-6 showed only a tendency to be lower than without IL-6 (*p* = 0.06) (Figure 5c). In fact, cells cultured with IL-6 might have stimulated a comparable environment to cells found in RA patients at baseline, characterized by higher IL-6 levels. Plasmatic levels of IL-35 increased after TCZ (t6 = 331.0 ± 425.2 pg/mL; *p* = 0.005). Additionally, IL-35 levels after treatment positively correlated with Treg CD39-CD73- cells (r = 0.431, *p* = 0.04). On the other hand, TCZ treatment did not increase plasmatic IL-10 levels (t6 = 239.4 ± 275.8 pg/mL).

### 3.6. The In Vitro Role of IL-6 in Influencing the Capacity of Effector T Cells to Produce IFNγ

**Figure 6:**
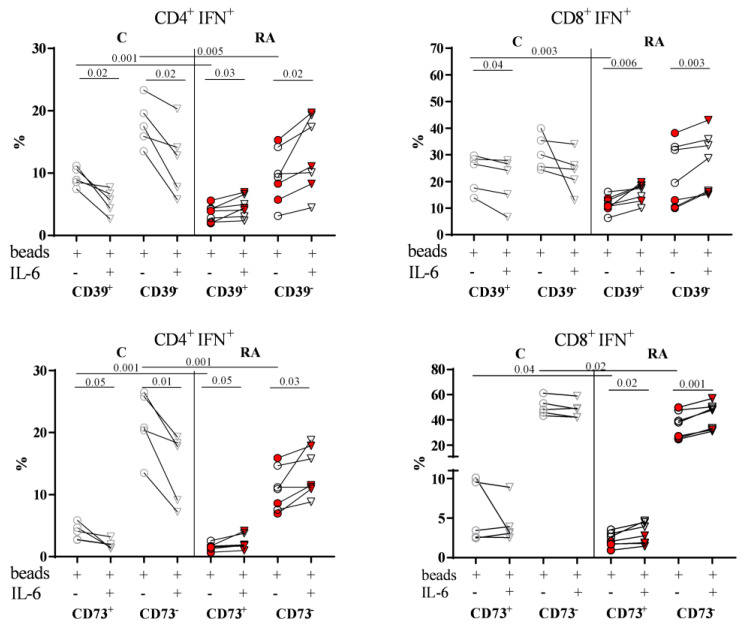
Effects of stimulation on CD39 and CD73 expression, and IFNγ production in CD4^+^ T and CD8^+^ T cells. The PBMCs from Cs (gray symbols) and RA patients (black symbols) were cultured for 72 h with beads and with (triangles) or without IL-6 (dots). The percentages of IFNγ-producing cells in CD4^+^ T (CD4^+^IFN^+^) and CD8^+^ T cells (CD8^+^IFN^+^) were categorized into two groups based on their expression of CD39 (CD39^+^ and CD39^−^) or CD73 (CD73^+^ and CD73^−^). In all plots, symbols colored in red correspond to NR-RA patients. Paired *t*-tests were applied in comparisons between subsets from C or RA patients and unpaired *t*-test to compare equivalent subsets in C with RA patients.

We next compared the percentage of IFNγ-producing CD4^+^ and CD8^+^ effector T cells (CD4^+^IFN^+^ and CD8^+^IFN^+^) within PBMCs from both RA patients and Cs, activated with or without IL-6 (Figure 6). When we examined CD39^+^ and CD73^+^ cells after activation, we observed a significantly higher percentage of IFNγ-producing CD4^+^ T and CD8^+^ T cells in Cs than in RA patients in cultures without IL-6 (Figure 6).

When we compared the activation with and without IL-6 (Figure 6), distinct patterns emerged between C and RA patients. In Cs, we found a significantly lower percentage of IFNγ-producing CD4^+^ T cells in cultures with IL-6, irrespective of CD39 expression (*p* = 0.02 in CD39^+^ subset and CD39^−^ subset) and CD73 expression (*p* = 0.05 in CD73^+^ subset and *p* = 0.01 in CD73^−^ subset). However, in RA patients, we observed a significantly higher percentage in cultures with IL-6, regardless of CD39 expression (*p* = 0.03 in CD39^+^ subset and *p* = 0.02 in CD39^−^ subset) and CD73 expression (*p* = 0.05 in CD73^+^ subset and *p* = 0.03 in CD73^−^ subset). For IFNγ-producing CD8^+^ T cells of RA patients, results mirrored those of CD4^+^ T cells, as the percentage was higher in cultures with IL-6, irrespective of CD39 expression (*p* = 0.006 in CD39^+^ subset and *p* = 0.003 in CD39^−^ subset) and CD73 expression (*p* = 0.02 in CD73^+^ subset and *p* = 0.001 in CD73^−^ subset). In contrast, only in the CD39^+^ subset of Cs was the percentage of IFNγ-producing CD8^+^ T cells lower in cultures with IL-6 (*p* = 0.04). Despite having a limited number of samples in this analysis, we did find differences between R-RA and NR-RA patients (red points in Figure 6).

To determine the effector capacity of CD4^+^ and CD8^+^ T cells, we assessed the Treg and Th17 function indirectly by measuring the levels of IL-10 and IL-17 in culture supernatants. We found that both cytokines tended to be higher in RA than in C cultures without IL-6 (IL-10: C = 324.3 ± 157.1, and RA = 518.1 ± 300.7; *p* = 0.08, and IL-17: C = 1565 ± 788.4, and RA = 3973 ± 2538; *p* = 0.06). No differences were found between cultures with and without IL-6. When we compared NR-RA and R-RA patients, three NR-RA patients presented higher levels of IL17 than four R-RA patients, whereas IL-10 levels were comparable in R-RA and NR-RA patients.

## 4. Discussion

In the current study, we have found different patterns of expression of CD39 and CD73 on different cell types from RA patients and Cs. In addition, the frequency of certain subsets based on CD39 and CD73 expression within CD4^+^ and CD8^+^ T cells and B cells exhibited correlations with disease activity indexes. In R-RA patients at baseline, we found that the percentage of certain subsets showed correlations with disease activity indexes, indicating potential associations between immune cell subsets and TCZ response. The in vitro experiments conducted to understand the role of IL-6 in modulating CD39 and CD73 expression revealed distinct responses between cells from RA patients and Cs.

We observed a lower percentage of CD8^+^CD39^−^CD73^+^ cells in RA patients compared to Cs. However, when comparing JIA patients with controls, Botta et al. [25] did not observe significant differences in CD73^+^ cells within CD8^+^ T cells. Several distinctions might account for these discrepancies. Notably, we specifically analyzed CD73^+^ cells that do not express CD39, a differentiation from Botta’s study, which included all CD73^+^ cells. Our analysis was performed on freshly stained peripheral blood CD8^+^ T cells instead of PBMCs, potentially leading to variations due to density separation. However, this methodological difference is likely not a major influence, as we found no significant differences in CD39 or CD73 expression on CD4^+^ T and CD8^+^ T cells obtained from fresh PB or PBMCs. Another differentiating factor is that our patient population was older than the JIA patients in Botta’s study, and since CD73 expression tends to decrease with age [26], this could contribute to divergent results.

We also observed a negative correlation between the percentage of CD8^+^CD39^−^CD73^+^ cells and disease activity indexes. This is consistent with patients having JIA, where reduced CD73 expression on synovial CD8^+^ T cells and B cells was reported, correlating with disease severity [25]. This is also in line with the findings of Ahmadi et al., who found that CD8^+^CD73^−^ secreted more proinflammatory cytokines and cytotoxic effector molecules [27]. Given the role of CD73 in adenosine production and the established correlation between CD73 expression in PB and synovial fluid [28], it is conceivable that the observed lower levels of CD8^+^CD39^−^CD73^+^ in RA patients might result in reduced adenosine production and a diminished capacity to regulate inflammation.

We confirmed a lower percentage of Treg cells in RA patients [29], with the most affected subsets being CD39^+^CD73^+^ and CD39^−^CD73^−^. With our current approach, we could not determine the suppressive function of these subsets. However, we found a positive correlation between Treg CD39^−^CD73^−^ cells with IL-35, an anti-inflammatory cytokine. Also, Dwyer et al. showed that the lack of CD39 expression on Tregs increments IL-10 and IFNγ production [30]. The low percentage of Treg CD39^−^CD73^−^ cells could indicate an impaired Treg differentiation, as RA patients often exhibit an imbalanced ratio between Treg and Th17 cells [31]. Moreover, we found that the percentage of Treg CD39^+^CD73^+^ cells tended to negatively correlate with DAS28, aligning with other authors who have reported a negative correlation between Treg frequency and DAS28 [29]. Notably, CD8^+^ T cells are a primary source of soluble CD73, which interacts with CD39-expressing Tregs to facilitate adenosine production [32]. Therefore, the decreased levels of both CD8^+^CD39^−^CD73^+^ and Treg CD39^+^CD73^+^ cells work in parallel to contribute to the impaired regulation of inflammation associated with RA.

In our investigation, we could not establish an association between Treg subsets delineated by the presence of CD39 and CD73 markers and the ATP–adenosine pathway, as indicated by the levels of ATP and ADP. We opted not to directly measure adenosine in plasma due to its inherent instability [33,34]. The lack of correlation with T regulatory cells (Tregs) could potentially be attributed to the expression of CD39 and CD73 in other cellular populations. These additional cell types may participate in the conversion of ATP, thereby contributing to the observed absence of association.

Our findings imply that certain subsets expressing CD39 and/or CD73 are associated with pathways modified by TCZ treatment, as evidenced by the post-treatment changes observed. The effects of MTX treatment have already been seen, as expected by the implication of CD39 involvement in the mechanism of action of MTX [13,14,15]. These TCZ-induced modifications appear to be geared towards regulating proinflammatory conditions in RA patients, with potentially heightened effectiveness in responder RA patients. In regard to CD8^+^CD39^−^CD73^+^ cells, we observed that the percentage of this subset exhibited a negative correlation with DAS28 in R-RA, but not in NR-RA patients. We cannot dismiss the possibility that the lack of statistically significant correlations in NR-RA patients could be due to the smaller number of patients displaying poor responses to treatment. It is worth noting that the percentage of this subset remained unchanged with TCZ treatment, suggesting that this cell population might be closely linked to RA pathology and could be less influenced by IL-6 or other pathways that TCZ treatment may alter. To validate this speculation, further experiments involving responders and non-responders to alternative therapies should be conducted.

TCZ is known to elevate Treg cell levels, and in line with this [35], we observed a specific increase in Treg CD39^−^CD73^−^ cells following treatment. Although this subset does not directly contribute to adenosine production, it could potentially act as a source of Treg-expressing CD39 cells with suppressor activity after activation [36]. Our results did not match those of Thiolat et al., who found after 3 months of anti-IL-6R treatment, there was a higher frequency of CD39^+^ Treg cells in responders vs. non-responders. It is possible that the two cohorts had differences in previous treatments and these ones can have an effect on the expression of CD39 [17]. Conversely, TCZ treatment led to an increase in the percentage of CD8^+^CD39^+^CD73^+^ cells, but notably only in NR-RA patients. While findings concerning CD39-expressing CD8^+^ T cells are controversial, some evidence suggests that their regulatory functions resemble those of Tregs [37]. Consequently, the elevated levels of CD8^+^CD39^+^CD73^+^ cells may indicate an ineffective attempt to regulate inflammation in NR-RA patients.

We observed that TCZ treatment led to a decrease in the percentage of B CD39^+^CD73^−^ cells in most R-RA patients. However, when analyzing RA patients treated with MTX, Zacca et al. observed an increase in the expression levels of CD39 on B cells among most good responders [16], without altering the percentages of B cell subsets. Our findings, in conjunction with Zacca’s study, suggest that different treatments can have varying effects on B cells [26,28]. On the other hand, B-cell subsets have been described to contribute to disease pathogenesis by influencing the expansion of Tregs, and we have observed the coexistence of downregulated B CD39^+^CD73^−^ with an increase in Treg CD39^+^CD73^−^ cells in responder patients [38].

At least in a short in vitro culture, we demonstrated that IL-6 does not alter the effect of activation on CD39 or CD73 expression in CD4^+^ T or CD8^+^ T cells. This result aligned those reported by Waldman et al., but it is apparently in contrast with the findings of Chalmin et al., who showed that IL-6 induced CD39 [39,40]. However, these latter authors explored in vitro Th17 cells generated with IL-6 plus TGF-β. This discrepancy between their findings and ours lies in the cell source: they utilized naïve T cells from mouse tissue and we employed PBMCs from human peripheral blood. Expectedly, IL-6 does modify the effector capacity of CD4^+^ T and CD8^+^ T-cell subsets, affecting their IFNγ production [41,42]. We confirmed that IFNγ-producing CD4^+^ T and CD8^+^ T cells were more prevalent in CD39^−^ or CD73^−^ subsets of CD4^+^ T and CD8^+^ T cells in controls and patients [25]. We observed disparate in vitro effects of IL-6 on cells from C and RA patients that may result from previous different conditions. Sustained elevated IL-6 levels in RA patients may induce their CD4^+^ T and CD8^+^ T cells to heighten their effector capacity when stimulated in the presence of IL-6 [43]. Conversely, populations obtained from Cs, not previously exposed to elevated IL-6 levels, exhibited a significant reduction in the effector capacity of CD4^+^ T cells when cultured in the presence of IL-6. Although we had limited samples, we compared the effector cells between R and NR patients. We did not find significant differences between the two groups of patients, but we cannot reach definitive conclusions until we have mop samples in each group. The interplay mechanism between IFNγ and IL-6 remains unclear [44] and could differ based on factors such as the balance between these molecules or the cellular environment.

We acknowledge that despite the results and implications, our study has limitations. The primary limitation lies in the restricted number of RA patients included, particularly when categorizing patients into R-RA and NR-RA groups. A larger patient cohort in a validation study would lead to more robust and statistically significant conclusions establishing subgroups of patients according to previous treatments. The secondary limitation concerns the absence of information regarding other molecules involved in purinergic signaling, which could be important for understanding the significance of CD39 and CD73 in RA pathophysiology. The third limitation is that in using this approach, we were unable to study the functionality of each subset. To minimize this limitation, we have included the association of specific effector subsets with IFNγ production and with the levels of plasmatic or supernatant cytokines.

Overall, the study provides insights into the dynamic nature of immune cell subsets and their potential relevance in RA pathogenesis and treatment. Specifically, it emphasizes the dynamic nature of CD39 and CD73 expression on lymphocytes, suggesting potential changes dependent on both time and context and implying a role in the altered immunoregulatory processes underlying RA pathophysiology.

## Figures and Tables

**Table 1:** Demographic characteristics of RA patients and Cs and clinical characteristics of RA patients. We applied an unpaired Student’s *t*-test (*) or χ^2^ (#).

	RA (n = 35)			C (n = 15)			
	n	Mean	SD	n	Mean	SD	*p*
Gender (women)	28			12			ns (#)
ACPA^+^	28						
RF^+^	23						
age (years)		**60.3**	12.9		**56.2**	12.1	ns (*)
DAS28		**5.4**	1.2				
SDAI		**29.2**	11.3				
CDAI		**27.4**	10.8				
ESR (mm/h)		**39.6**	22.4				
CRP (mg/dL)		**1.7**	1.6				
Ig G (mg/dL)		**1182.1**	280.7				
Ig A (mg/dL)		**294.1**	178.8				
Ig M (mg/dL)		**167.3**	179.4				
Monocytes (10^3^/µL)		**0.7**	0.3				
Neutrophils (10^3^/µL)		**6.4**	2.9				
Lymphocytes (10^3^/µL)		**1.9**	0.8				
Platelets (10^3^/µL)		**293.0**	80.9				

## Data Availability

Experimental data can be found in the tables and figures presented in this manuscript.

## Supplementary Materials

The following supporting information can be downloaded at https://www.mdpi.com/article/10.3390/biom14010001/s1. Figure S1: Comparison of Pearson’s correlation coefficient (r) values between the C and RA corre-lation matrices; Figure S2: Correlations in R-RA and NR-RA; Table S1: Demographic and clinical characteristics of R and NR-RA patients.
