# Supplementary material for: Altered CD39 and CD73 Expression in Rheumatoid Arthritis: Implications for Disease Activity and Treatment Response
Source: Biomolecules. 2023 Dec 19;14(1):1. doi: 10.3390/biom14010001 (PMC10813161; doi:10.3390/biom14010001)
Supplement: Supplementary file 1 [file biomolecules-14-00001-s001.zip › Table S1.pdf]

| | | R-RA (n=20) | | | NR-RA (n=10) | | |
|---|---|---|---|---|---|---|---|
| | n | **Mean** | SD | n | **Mean** | SD | p |
| Gender (women) | 19 | | | 4 | | | 0.002 (#) |
| ACPA + | 17 | | | 7 | | | ns (#) |
| RF+ | 15 | | | 6 | | | ns (#) |
| age (years) | | **57.6** | 13.1 | | **61.45** | 11.4 | ns (*) |
| DAS28 | | **5.38** | 1.1 | | **5.34** | 1.3 | ns (*) |
| SDAI | | **28.06** | 12.1 | | **30.41** | 10.7 | ns (*) |
| CDAI | | **26.57** | 11.8 | | **28.12** | 9.6 | ns (*) |
| ESR (mm/h) | | **37.48** | 19.0 | | **40.8** | 28.4 | ns (*) |
| CRP (mg/dl) | | **1.48** | 1.1 | | **2.28** | 2.2 | ns (*) |
| IgG (mg/dl) | | **1197** | 342.7 | | **1161** | 222.2 | ns (*) |
| IgA (mg/dl) | | **333.9** | 219.6 | | **250.6** | 143.6 | ns (*) |
| IgM (mg/dl) | | **184.3** | 242.6 | | **139.5** | 59.56 | ns (*) |
| Monocytes ($10^3/\mu l$) | | **0.66** | 0.28 | | **0.71** | 0.18 | ns (*) |
| Neutrophils ($10^3/\mu l$) | | **5.66** | 3.45 | | **7.16** | 1.61 | ns (*) |
| Lymphocytes ($10^3/\mu l$) | | **1.95** | 0.75 | | **1.88** | 0.77 | ns (*) |
| Platelets ($10^3/\mu l$) | | **288.6** | 83.93 | | **282.7** | 63.84 | ns (*) |
