# Supplementary figures and images for: Altered CD39 and CD73 Expression in Rheumatoid Arthritis: Implications for Disease Activity and Treatment Response
Source: Biomolecules. 2023 Dec 19;14(1):1. doi: 10.3390/biom14010001 (PMC10813161; doi:10.3390/biom14010001)

C

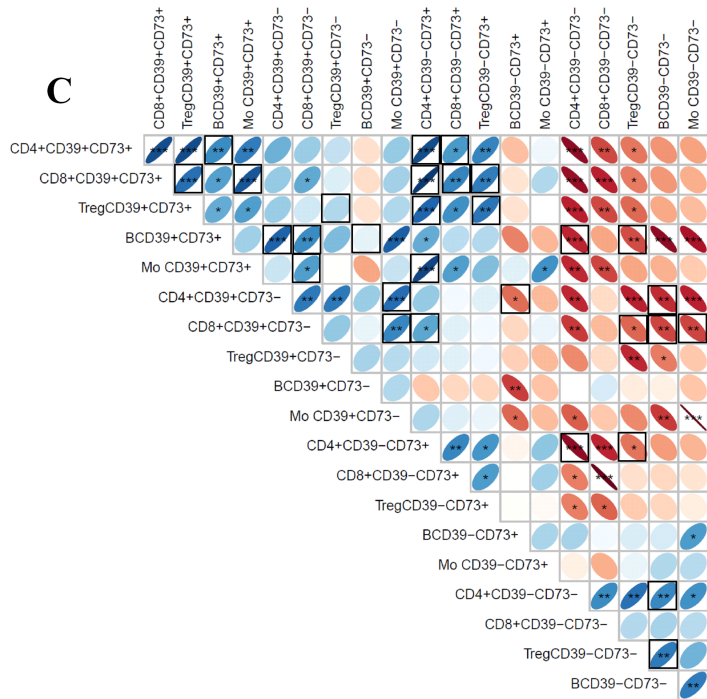

RA

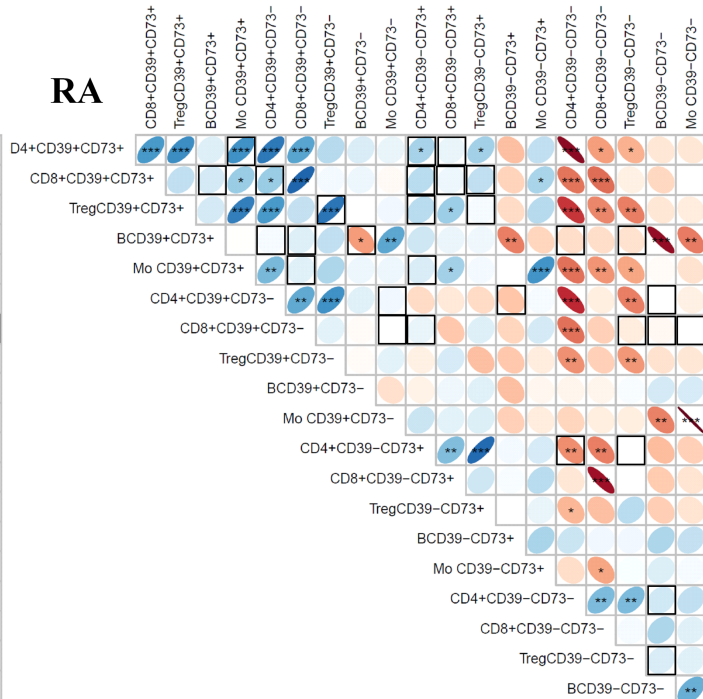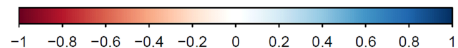

Supplement: Supplementary file 1 [file biomolecules-14-00001-s001.zip › Figure S1.pdf]

**a.**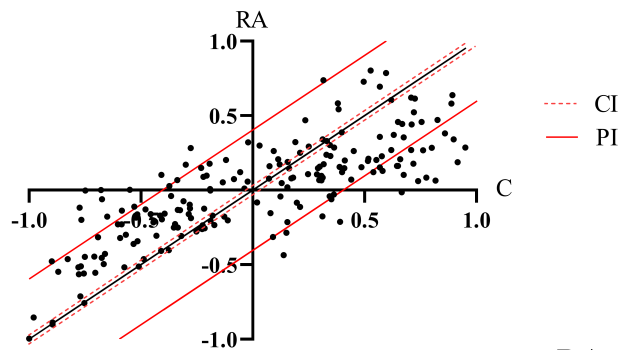**b.****C****RA**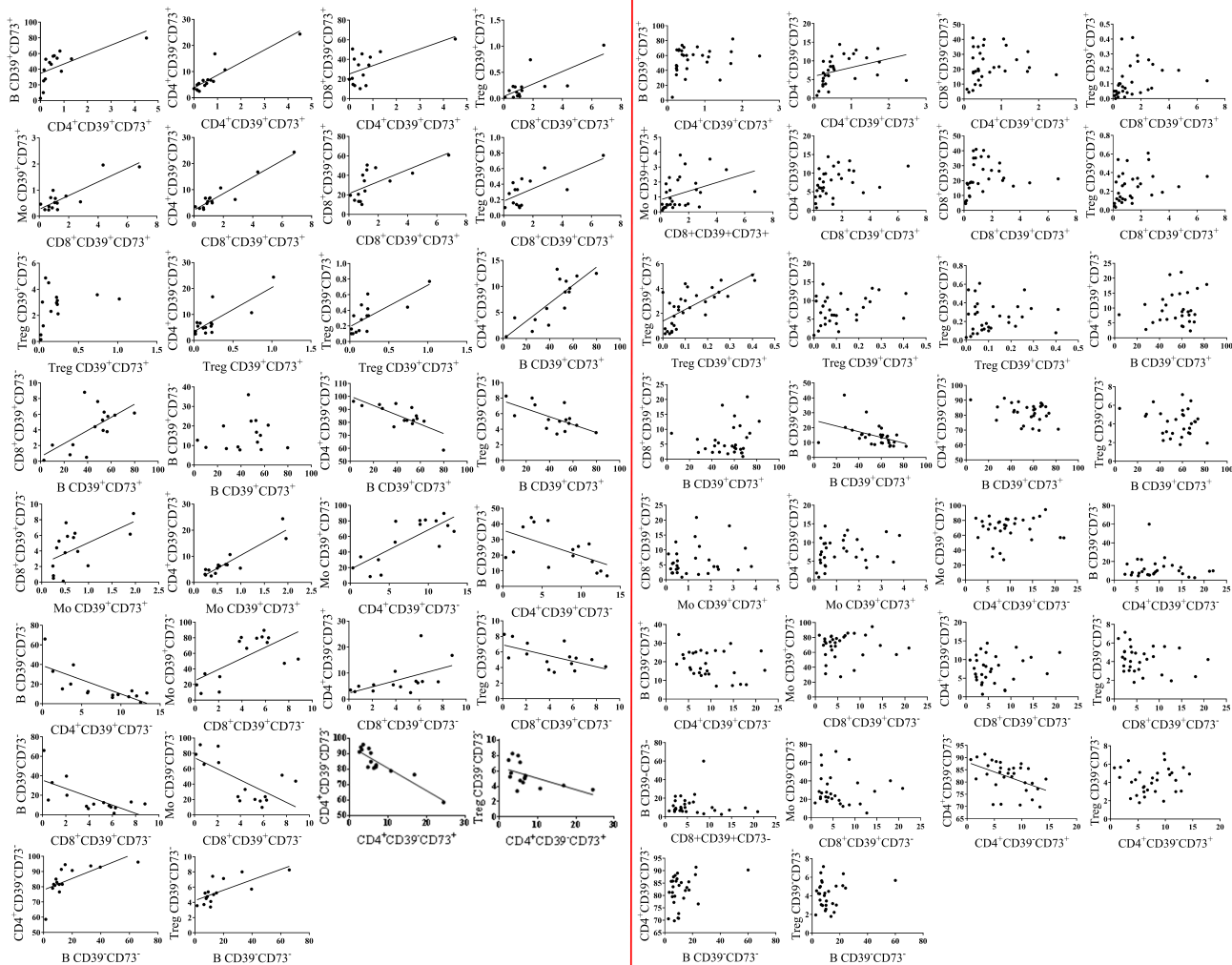

Supplement: Supplementary file 1 [file biomolecules-14-00001-s001.zip › Figure S2.pdf]
